# Genes Identification, Molecular Docking and Dynamics Simulation Analysis of Laccases from *Amylostereum areolatum* Provides Molecular Basis of Laccase Bound to Lignin

**DOI:** 10.3390/ijms21228845

**Published:** 2020-11-22

**Authors:** Ningning Fu, Jiaxing Li, Ming Wang, Lili Ren, Youqing Luo

**Affiliations:** 1 Beijing Key Laboratory for Forest Pest Control, Beijing Forestry University, Beijing 100083, China; funingning2012@sina.com (N.F.); jiaxingli2020@163.com (J.L.); 13020028768@163.com (M.W.); 2 Sino-French Joint Laboratory for Invasive Forest Pests in Eurasia, Beijing Forestry University—French National Research Institute for Agriculture, Food and Environment (INRAE), Beijing 100083, China

**Keywords:** *Amylostereum areolatum*, *Sirex noctilio*, laccases, molecular docking and dynamics simulation, protein-ligand interaction

## Abstract

An obligate mutualistic relationship exists between the fungus *Amylostereum areolatum* and woodwasp *Sirex noctilio*. The fungus digests lignin in the host pine, providing essential nutrients for the growing woodwasp larvae. However, the functional properties of this symbiosis are poorly described. In this study, we identified, cloned, and characterized 14 laccase genes from *A. areolatum*. These genes encoded proteins of 508 to 529 amino acids and contained three typical copper-oxidase domains, necessary to confer laccase activity. Besides, we performed molecular docking and dynamics simulation of the laccase proteins in complex with lignin compounds (monomers, dimers, trimers, and tetramers). AaLac2, AaLac3, AaLac6, AaLac8, and AaLac10 were found that had low binding energies with all lignin model compounds tested and three of them could maintain stability when binding to these compounds. Among these complexes, amino acid residues ALA, GLN, LEU, PHE, PRO, and SER were commonly present. Our study reveals the molecular basis of *A. areolatum* laccases interacting with lignin, which is essential for understanding how the fungus provides nutrients to *S. noctilio.* These findings might also provide guidance for the control of *S. noctilio* by informing the design of enzyme mutants that could reduce the efficiency of lignin degradation.

## 1. Introduction

The European woodwasp, *Sirex noctilio* Fabricius (Hymenoptera; Symphyta; Siricidae), is an important pest that causes considerable economic and ecological damage globally [1]. It has been recognized by the North American Plant Protection Organization (NAPPO) and the United States Department of Agriculture (USDA) as an invasive species posing “extreme risk” [1,2]. *S. noctilio* attacks *Pinus* species distributed across six continents, including *Pinus pinaster*, *P. radiata*, *P. elliottii*, *P. sylvestris*, and *P. taeda* [3,4,5,6,7]. In China, *S. noctilio* was first discovered in July 2013 in Daqing, Heilongjiang Province, and was subsequently added to the “National List of Forest Hazardous Pests” [8]. To date, *S. noctilio* has spread to 23 cities in northeast China, severely damaging local *Pinus sylvestris* var. *mongolica* plantations, and causing substantial economic and ecological losses [9,10].

*Amylostereum areolatum* (Fr.) Boidin (Basidiomycotina: Corticiaceae), as a fungal symbiont of *S. noctilio*, has a strict obligate dependency mutualism with the woodwasp [8]. During oviposition, female woodwasps inject symbiotic fungus and phytotoxic venom together with an egg into the woody stems of the host pine tree [11,12,13]. The phytotoxic venom rapidly weakens host plant resistance by inhibiting water transfer, which allows *A. areolatum* to spread [14,15,16]. Subsequently, the symbiotic fungus kills the host tree cells by blocking and cavitation of the xylem channel [17]. The fungus relies on female wasps to disperse and inoculate into new hosts, as the females carry the oidia inside their mycangia and inject them during oviposition [8]. In turn, like most wood-decay fungi, *A. areolatum* produces a large number of enzymes to digest all compounds of pine wood in xylem tissues, which provides a primary source of nutrition for the woodwasp larvae [16,18,19,20,21]. The foraging patterns (foraging in areas of most significant fungal symbiont enzyme activity), mandibular morphology (specialization of mandibles for shearing xylem and squeezing out liquid extracts from decomposed wood) and internal anatomy (limited gut lumen volume and lack of specialized fermentation chambers, and almost no woody tissue in the gut) of *S. noctilio* support the hypothesis that the fungal symbiont acts as an “external rumen”, digesting woody fibers into starch and reduced sugars for the larvae to absorb [20]. Bordeaux suggests that there is little peroxidase activity in the suitable growth medium for *A. areolatum*, and laccase is the sole enzyme with measurable phenoloxidase activity. In addition, a 75 kDa laccase protein is partially-purified in this system [22]. Some studies suggest that almost all white-rot fungi show extracellular phenoloxidase activity, and laccase and peroxidase in these phenoloxidases are the most important enzymes involved in decomposing lignin [22]. Therefore, laccase may play an important role in the nutritional mutualisms between *S. noctilio* and *A. areolatum*, but so far, no laccase genes of the symbiotic fungus have been reported.

Laccases (EC 1.10.3.2) are blue multi-copper enzymes, which can use the unique redox ability of copper ions to catalyze the oxidation of various aromatic substrates concomitantly with the reduction of molecular oxygen to water [23,24]. Studies have found that laccase enzymes are widely distributed in nature, having been detected in higher plants, fungi, insects and bacteria [25,26,27]. In fungi, they are involved in fungal pathogenesis, spore formation, pigmentation, fruiting body formation, and melanin formation, amongst others [28,29,30,31]. In addition, the laccase-mediated degradation of lignin polymers is one of the most important functions of fungal laccases, since these enzymes can use oxygen as an oxidant to help degrading lignin [32,33]. Fungal laccases generally contain two copper centers, which are responsible for electron transfer during the redox reaction. The two copper centers are the mononuclear center T1 with one copper atom (type-1 Cu) responsible for the blue color, and the trinuclear cluster (T2/T3) consisting of one copper atom (type-2 Cu) and two coupled copper atoms (type-3 Cu), respectively [23,34]. Laccases catalyze the substrate’s oxidation through the concerted action of these four copper ions in electron transfer. Compared with lignin peroxidase, the catalytic process of laccase does not require the participation of H_2_O_2_ [35]. So, this enzyme has an advantage in catalyzing the degradation of lignin.

Fungal laccase can directly oxidize phenolic subunits, such as ortho, *para*-diphenols, aminophenols, polyphenols, polyamines, and aryl diamines due to its high redox potential [36,37,38,39]. It also can use the laccase mediator system to oxidize the non-phenolic subunits of lignin, thereby playing a role in the degradation of lignin [40]. Laccases generally show low substrate specificity, and the range of oxidized substrates can vary between laccases [41]. It is also proposed that the overall three-dimensional (3D) structure of laccase can lead to changes in the microenvironment of the enzyme’s active site. Furthermore, the structures of lignin model compounds affect the oxidation rate of laccase [42]. Therefore, it is necessary to understand how laccase interacts with lignin compounds. Molecular docking and molecular dynamics simulation are reliable methods to explore protein-ligand interactions at the atomic and molecular levels [43].

Fungal laccases usually exist in gene family, and the laccase isozymes display diverse physicochemical characteristics [22]. However, no laccase gene of *A. areolatum* has been reported. In this study, we identified, cloned, and characterized 14 laccase genes of *A. areolatum*. We then used molecular docking and molecular dynamics simulation to study the interactions between these laccases and different lignin compounds (monomers, dimers, trimers, and tetramers). We found that five laccases had low binding energies with all lignin model compounds and three of them could maintain stability when binding to these lignin compounds. Both hydrogen bonds and hydrophobic interactions played important roles in the binding of these complexes. Our results would enhance our understanding of how *A. areolatum* enzymes catalyze the biodegradation of lignin, to provide nutrition for the growing larvae of *S. noctilio*. Ultimately, we have expanded our understanding of the symbiotic relationship between *A. areolatum* and *S. noctilio*.

## 2. Results

### 2.1. Laccase Activity of A. areolatum

**Figure 1:**
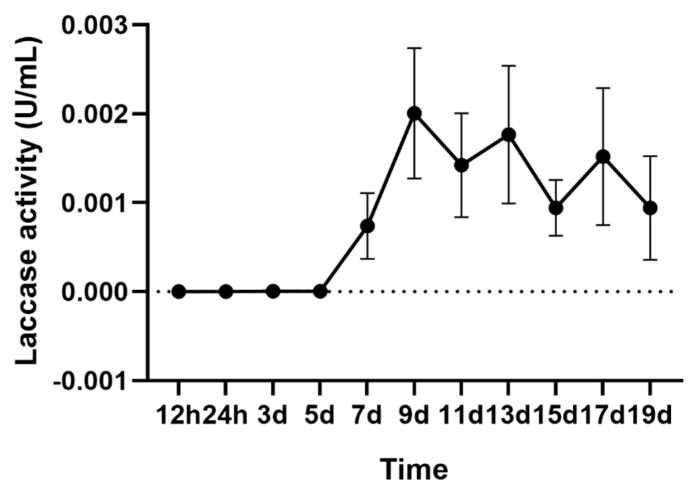
The laccase activity of *A. areolatum* from 1–19 days.

The laccase activity of *A. areolatum* grown in Potato Dextrose Broth (PDB) medium at 28 °C for 19 days was measured every two days. A linear increase in activity was observed after five days, and maximal activity was recorded after nine days (0.002 U/mL). Subsequently, the activity of laccase fluctuated within 0.001–0.002 U/mL and gradually decreased (Figure 1).

### 2.2. Identification and Properties of Laccase Genes in A. areolatum

**Figure 2:**
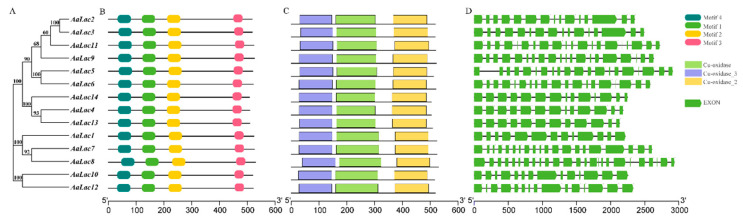
Phylogenetic trees, motif, domain and gene structure of *A. areolatum* gene family. (**A**) The phylogenetic tree of *AaLac* genes; (**B**,**C**) conserved motifs and domains of the AaLac proteins, different colors represent different motifs or domains. (**D**) exon-intron structures, exons are indicated by green boxes and introns by lines.

To identify potential laccase genes in *A. areolatum*, we performed BLASTp searches using protein sequences corresponding to laccases in related organisms, and searched three Hidden Markov Model (HMM) profiles within the genome of *A. areolatum* (GenBank accession number: SAXG00000000 and the BioProject accession number PRJNA513942) [44]. A fourteen-member laccase multigene family was identified (Table 1). The length of the nucleotide sequences of the identified laccase genes ranged from 2134 to 2910 base pairs, and genes contained 11 to 23 introns with an average length of 58 base pairs (Figure 2D). The laccase genes encoded proteins ranging from 508 to 529 amino acids with predicted molecular weights ranging from 54 kDa to 58 kDa. Theoretical isoelectric points of the 14 laccase proteins ranged from 4.20 to 6.12. Except for AaLac3, all laccase proteins had a signal peptide of 18 to 33 amino acids and no transmembrane region (Figure S1), which indicated that these were secretory proteins. In addition, the identified proteins were found to be stable, with instability index values of between 28.80 and 38.62 for all proteins except for AaLac7 and AaLac8 (which had values of 44.01 and 42.38, respectively). We also analyzed the number of cysteine residues present and the prevalence of disulfide bridges in the laccase protein sequences. All laccase proteins contained five cysteine residues (except AaLac10 and AaLac12) and at least one disulfide bridge (except AaLac6). Analysis of the secondary structures of the fourteen proteins revealed the presence of α-helices (6.58%–13.08%), extended strands (29.39%–31.91%), β-turns (6.29%–8.46%), and random coils (49.12%–54.49%) (Table S1).

### 2.3. Gene Structure and Sequence Alignment of Laccase Genes in A. areolatum

To investigate their structural diversity, we analyzed the exon-intron organization of the identified laccase genes, and searched for conserved motifs and domains based on the phylogenetic tree of all laccase alignments (Figure 2). Overall, the phylogenetic tree and exon-intron structures indicated a close relationship between laccase genes with the same intron phase. For example, a similar number of introns were found in *AaLac5* and *AaLac6*, as well as *AaLac4*, *AaLac13*, and *AaLac14*. However, despite being highly related (bootstrap value of 100%), a small proportion of genes exhibited different intron-exon organizations. For example, *AaLac7* and *AaLac8* have similar intron-exon structures, but they differ significantly from the structure of the highly related *AaLac1*.

**Figure 3:**
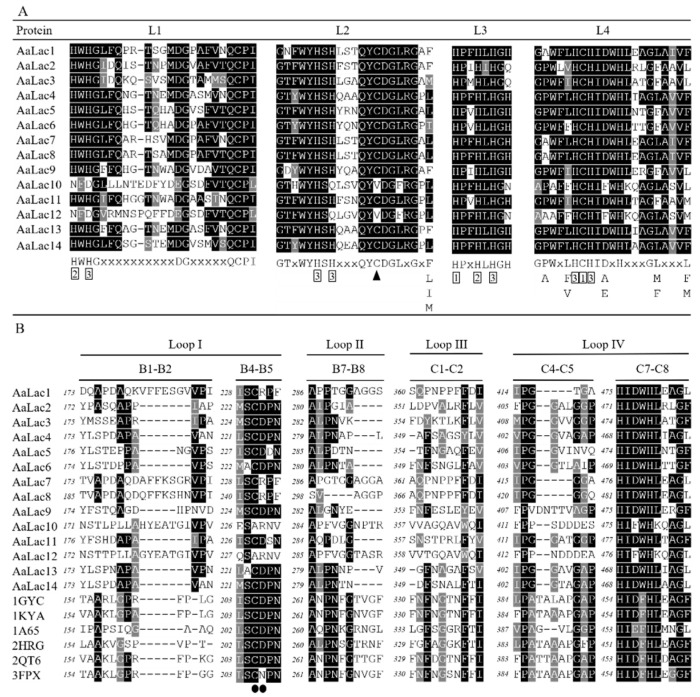
The signature sequences and postulated substrate binding loops of *A. areolatum* laccase. (**A**) The signature sequences (L1–L4) of AaLac1–AaLac14. The typically conserved ten histidine and one cysteine residues of the copper-binding centers are numbered according to the copper type (1, 2, and 3 for type-1, type-2, and type-3, respectively) they bind [50]. The black triangle marks the cysteine (C) residue in signature sequence L2, which is always present in classical laccases but not in Mco1 and Fet3 ferroxidases [51,52]. (**B**) The substrate binding loops (I–IV) of AaLac and known three-dimensional structures. The black circle underneath the sequences indicate residues in the β-hairpin loop B4–B5, which in the classical laccases are a highly conserved cysteine (C) and either an aspartic acid (D) or a glutamic acid (E) existed in *T. versicolor* LacIIIb to contact with organic substrates [53]. The identical and similar residues are represented by black and grey shading.

Alignment of the deduced amino acid sequences of *A. areolatum* laccases showed that these proteins appear to be typical laccases; we identified motif sequences corresponding to fungal laccase signature sequences L1–L4 (Figure 2B and Figure S2). Within the signature sequences were conserved ten histidines and one cysteine residue of the copper-binding centers (T1 and T2/T3) (Figure 3A). Interestingly, AaLac10 and AaLac12 lacked laccase signature sequence L1, specifically, the two conserved histidine residues, and had valines instead of cysteines in L2. The postulated substrate binding loops I–IV of *A. areolatum* were identified by comparison with the 3D-structures of laccase proteins from *Trametes versicolor* (Protein Data Bank (PDB) code: 1GYC; 1KYA) [45], *Coprinopsis cinerea* (PDB code: 1A65) [46], *Trametes trogii* (PDB code: 2HRG) [47], *Lentinus tigrinus* (PDB code: 2QT6) [48], and *Trametes hirsuta* (PDB code: 3FPX) [49]. Consistent with the phylogenetic analysis, we found that the substrate binding loops of AaLac1, AaLac7, AaLac8, AaLac10, and AaLac12 were significantly different from other sequences; neither an aspartic acid (D) nor a glutamic acid (E) were found in the β-hairpin loop B4–B5 (Figure 3B). Furthermore, AaLac10 and AaLac12 also lacked a cysteine residue in the loop B4–B5, which was found in all other *A. areolatum* laccases (Figure 3B). The conserved motifs of the AaLac proteins were identified using the Pfam database, which showed that three characteristic domains (Cu-oxidase, Cu-oxidase_2, and Cu-oxidase_3) were present in all *A. areolatum* laccase proteins (Figure 2C).

### 2.4. Phylogenetic Analysis of A. areolatum Laccases

**Figure 4:**
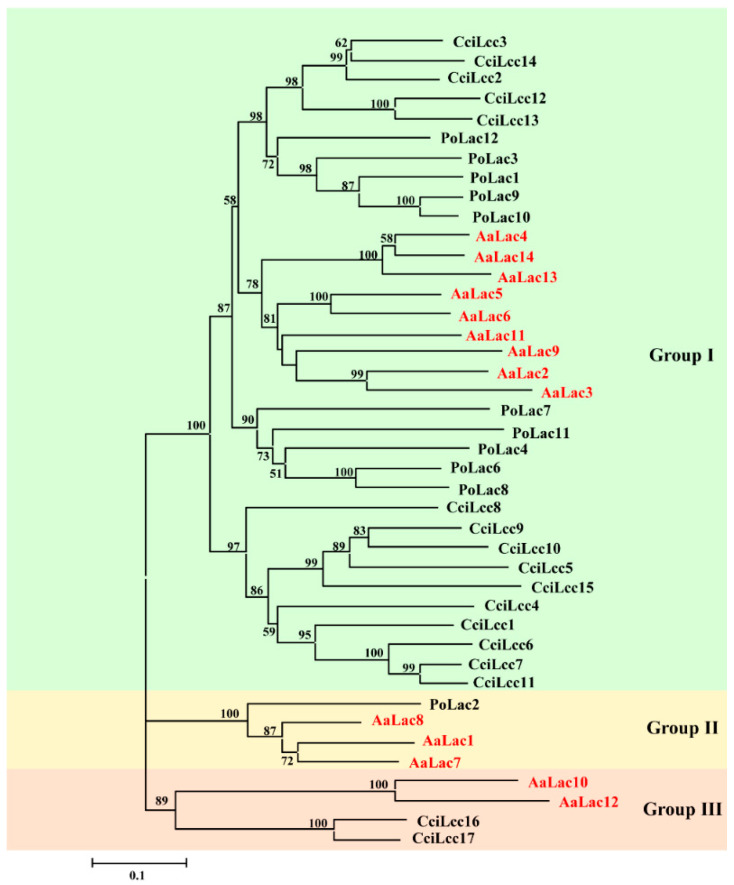
The phylogenetic tree of laccase proteins from *A. areolatum*, *C. cinerea* and *P. ostreatus*. The tree was constructed by MEGA 6.0 using NJ method with bootstrap replication of 1000 times. The laccases of *C. cinerea* (CciLcc1–CciLcc17 with GeneBank accession numbers DAA04506–DAA04522) and *P. ostreatus* (PoLac1 with JGI Protein ID 1043420; PoLac2 with JGI Protein ID 1067328; PoLac3 with JGI Protein ID 1102751; PoLac4 with JGI Protein ID 1077328; PoLac6 with JGI Protein ID 1113032; PoLac7 with JGI Protein ID 1077468; PoLac8 with JGI Protein ID 1106925; PoLac9 JGI with Protein ID 1089733; PoLac10 with JGI Protein ID 1089723; PoLac11 with JGI Protein ID 1043488; PoLac12 with JGI Protein ID 1094965).

A phylogenetic tree was constructed using the laccase sequences of *A. areolatum*, *C. cinerea*, and *P. ostreatus* (Figure 4). Based on the classification of laccase proteins in *C. cinerea* and *P. ostreatus*, the 14 AaLac proteinss were divided into three distinct groups: group I, group II, and group III. Group I contained the most AaLacs, which should belong to the laccases sensu stricto subfamiliy 1 defined by the *C. cinerea* enzymes. AaLac1, AaLac7, and AaLac8 displayed high protein homology with known laccases PoLac2 and were clustered in group II. AaLac10 and AaLac12 grouped with two closely related laccases CciLac16 and CciLac17, which might have both laccase and iron oxidase activities.

### 2.5. Homology Modeling and Validation

To identify appropriate templates for the laccase proteins, both PDB and SWISS-MODEL library BLAST searches were performed. Proteins with the highest similarity scores (ranging from 42.86%–65.06%) were selected as templates (Table 2). The 3D modeled protein structures of fungal laccase proteins had high QMEAN and GMQE scores, indicating the predicted structures were likely of high quality. These initial laccase models were then refined using GalaxyRefine web servers and validated by the Structure Analysis and Verification Server (SAVES). Ramachandran plots showed that all the refined models had almost 90% of residues in favorable regions and more than 99.5% of residues in the permissible areas (Table 2 and Table S2 and Figure S3). G-factor values were all greater than −0.5, which indicated that the distribution of torsion angles and covalent geometries within the models were reasonable. Similarly, in all of the generated models, we found more than 90% of residues had an average 3D-1D score > 0.2 and overall quality factor values > 82.5. Generally, the homology models (factor values > 50) were stable and reliable. ProSA analysis revealed that the Z-scores for all the models ranged between −8.62 and −7.16. Finally, we looked at the LGscore and MaxSub to assess the quality of the protein models. Scores greater than 3 (LGscore) or 0.5 (MaxSub) indicate good quality models. With the exception of AaLac10, all laccase structures achieved significant scores, with LGscores greater than 4 and MaxSub scores greater than 0.3. Altogether, these results revealed that the models obtained using homology modeling were acceptable and could be used for further study.

### 2.6. Molecular Docking of A. areolatum Laccases

**Figure 5:**
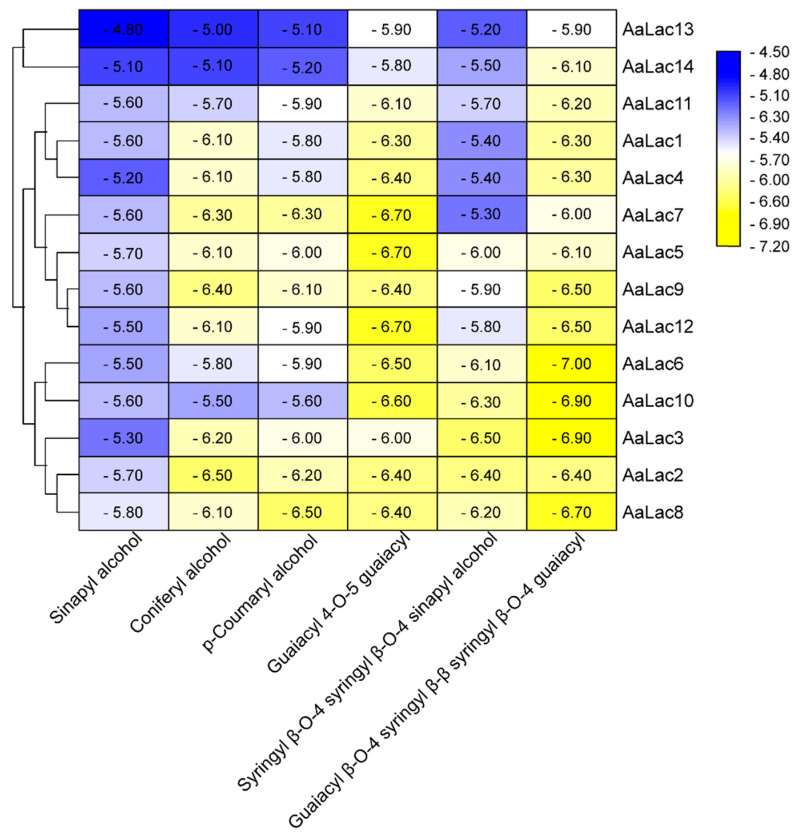
The minimum binding energy scores (kcal/mol) of *A. areolatum* laccases with lignin model compounds.

To better understand the molecular basis of *A. areolatum* laccases interacting with lignin, we performed molecular docking studies using the identified laccase protein sequences in combination with six different lignin model compound: sinapyl alcohol (SA), coniferyl alcohol (CA), *p*-coumaryl alcohol (CoA), guaiacyl 4-O-5 guaiacyl (dimer), syringyl β-O-4 syringyl β-O-4 sinapyl alcohol (trimer), and guaiacyl β-O-4 syringyl β-β syringyl β-O-4 guaiacyl (tetramer). When comparing the docking results between the lignin complexes and laccases, the most noticeable difference was the interaction energy, which ranged from −7.0 to −4.8 kcal/mol. We found the laccases, AaLac2, AaLac3, AaLac6, AaLac8, and AaLac10 had low binding energies with all lignin compounds. Furthermore, we observed the binding energies decreased with the increasing of ligand size; binding energies between laccases and tetramers were lower than that between laccases and monomers (although there were some exceptions to this rule in the case of the lignin trimer) (Figure 5).

**Figure 6:**
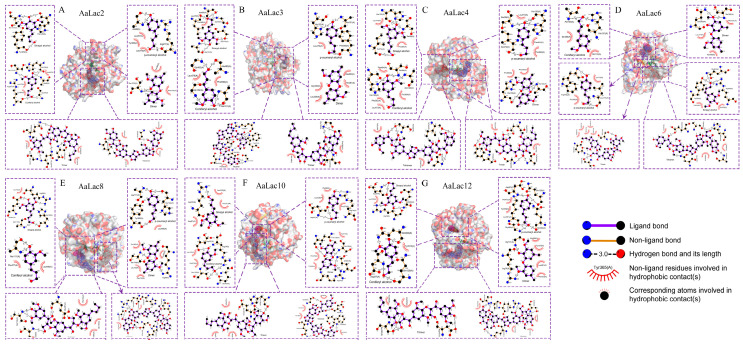
Interactions of *A. areolatum* laccase with lignin model compounds: sinapyl alcohol (SA), coniferyl alcohol (CA), p-coumaryl alcohol (CoA), guaiacyl 4-O-5 guaiacyl (dimer), syringyl β-O-4 syringyl β-O-4 sinapyl alcohol (trimer), and guaiacyl β-O-4 syringyl β-β syringyl β-O-4 guaiacyl (tetramer). (**A**) AaLac2; (**B**) AaLac3; (**C**) AaLac4; (**D**) AaLac6; (**E**) AaLac8; (**F**) AaLac10; (**G**) AaLac12.

Hydrogen-bond interactions, together with hydrophobic contacts, were found necessary to the interactions of lignin model compounds with Lac proteins. We investigated the molecular interactions for binding between laccases and lignin compounds and found that hydrogen bonding and hydrophobic interactions were present. We observed hydrogen bonds between the majority of laccase-lignin combinations, with the exception of SA-AaLac9 and AaLac13; CoA-AaLac13 and dimer-AaLac3, AaLac5, AaLac9, and AaLac14 (Figure 6 and Figures S4–S10). Among them, strong hydrogen bonds were formed between lignin model compounds and the laccase proteins AaLac2, AaLac3, AaLac4, AaLac6, AaLac8, AaLac10, and AaLac12 (Figure 6). Similarly, we found that the lignin model compounds formed hydrophobic interactions with amino acids around the laccase binding site. Only AaLac1 and AaLac2, in combination with SA, failed to form hydrophobic interactions (Table S3). When looking at the active sites of laccase proteins, we found that 18 amino acid residues, namely, ALA, ARG, ASN, ASP, GLN, GLU, GLY, HIS, ILE, LEU, LYS, PHE, PRO, SER, THR, TRP, TYR, and VAL, were involved in the hydrogen bonding and hydrophobic interactions of the docked complexes (Table S3). Interestingly, common amino acid residues ALA, GLN, LEU, PHE, PRO, and SER were present in the interactions between model compounds and laccases. The orientation of the ligand is important for acceptor-binding activity. Clearly, most laccase proteins (AaLac1, AaLac2, AaLac7, AaLac9, AaLac10, AaLac12, and AaLac14) showed similar binding patterns, monomers and dimer; trimer and tetramer bound to the same pocket in different conformations, respectively. In addition, we observed that in some laccases, monomers, dimer, and trimer (tetramer) bound at the same pocket, while the other tetramer (trimer) bound separately at different pocket (Figure 6 and Figures S4–S10).

### 2.7. Molecular Dynamics Simulation of Laccase with Lignin Model Compounds

**Figure 7:**
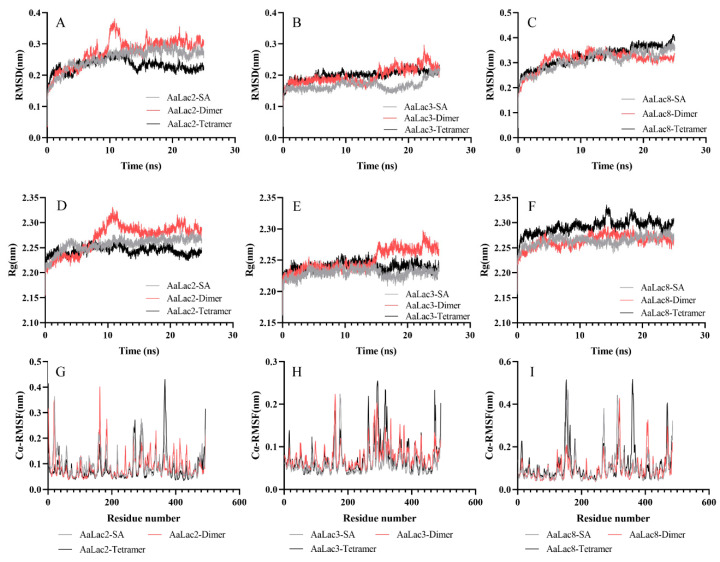
The variation of (**A**–**C**) root mean square (RMSD), (**D**–**F**) radius of gyration (Rg) and (**G**–**I**) root mean square fluctuation (RMSF) for AaLac2-SA, AaLac2-dimer, AaLac2-tetramer, AaLac3-SA, AaLac3-dimer, AaLac3-tetramer, AaLac8-SA, AaLac8-dimer, and AaLac8-tetramer.

Consistent with previous studies, we chose laccases having high binding efficiencies with all lignin compounds and three types of lignin model compounds, namely, SA, dimer, and tetramer to conduct molecular dynamics (MD) simulation. The root mean square (RMSD), the radius of gyration (Rg) and root mean square fluctuation (RMSF) were used to analyze the stability of the docked complexes. The RMSD plots showed that almost all docked complexes quickly tended to reach equilibrium and maintained an equilibrium between 15–25 ns during the simulation. It was noteworthy that AaLac3-tetramer docked complex was more stable than other structures (Figure 7B). Rg represents the compaction of the complex. The Rg plots showed that, except for the complex AaLac2-Dimer, the Rg of the analyzed structures did not fluctuate sharply during the MD simulation. The average Rg for the complexes of AaLac2-SA, AaLac2-dimer, AaLac2-tetramer, AaLac3-SA, AaLac3-dimer, AaLac3-tetramer, AaLac8-SA, AaLac8-dimer, and AaLac8-tetramer was 2.26, 2.24, 2.25, 2.23, 2.24, 2.26, 2.26, and 2.29 nm, respectively. Figure 7G–I showed the RMSF of the laccases Cα in 15–25 ns (equilibrium phase) of MD simulation. The Cα-RMSF value for AaLac2-SA, AaLac2-dimer and AaLac2-tetramer was highest in residue 162, 20 and 366, respectively. The higher flexibility of AaLac3 with lignin model compounds (monomer to tetramer) were in regions composed of residues 157–161, 173–181, and 468–491; 151–163, 261–270, 279–342, 356–369, and 374–378; 14–18, 156–162, 261–269, 280–324, 333–335, and 468–491. The maximum fluctuations for the docked complexes of AaLac8 with SA, dimer, and tetramer were found in residue 157, 319 and 360; and the key fluctuations for these structures occurred around the residues 144–167, 264–276, 302–335, and 461–485; 284–335, 401–425, and 460–475; 176–184, 353–376, and 460–479, respectively. In addition, we analyzed the average interaction energy of the docked structures during 15–25 ns molecular dynamic simulation. The average interaction energy of the complexes of laccase proteins AaLac2, AaLac3, and AaLac8 with the lignin model compounds (monomer to tetramer) were −28.19 kJ·mol^−1^, −138.12 kJ·mol^−1^, −235.78 kJ·mol^−1^, −3.16 kJ·mol^−1^, −78.67 kJ·mol^−1^, −231.99 kJ·mol^−1^, −114.22 kJ·mol^−1^, −138.99 kJ·mol^−1^, and −224.39 kJ·mol^−1^, respectively.

## 3. Discussion

Wood-feeding insects face many difficulties in obtaining nutrition from their chosen food source. A mutualistic relationship exists between the fungus *A. areolatum* and the woodwasp *S. noctilio* [8]; the introduction of *A. areolatum* into host trees by *S. noctilio* is necessary for fungal dispersion [1], whilst the degradation of wood tissues by *A. areolatum* is crucial for the survival of *S. noctilio* larvae as this provides essential nutrition [18].

Genome analysis of *A. areolatum* indicates that the fungus contains large amounts of Carbohydrate-Active Enzymes, which might be involved in degrading plant cell walls [44]. We detected laccase activity in *A. areolatum* cultures after five days of growth, and recorded maximum activity on the ninth day. This result was consistent with previously reported research in *A. areolatum*, which found that peak laccase activity was reached 8–10 days after inoculation [22]. Whilst, the extracellular laccase activity of *A. areolatum* remained relatively low throughout the incubation process, some inducers, likely SDS and EDTA, could significantly increase laccase activity [22]. Furthermore, Li et al. found that *S. noctilio* venom’s presence could significantly increase laccase activity in *A. areolatum* [8]. These results suggest that laccase activity can be enhanced by *S. noctilio* venom, resulting in an ideal environment for insect larvae’s growth.

We identified 14 laccase genes in the genome of *A. areolatum*. The results of gene structure analyses (Figure 2D) revealed similar exon-intron structures between most laccase genes clustered in the same group. The same pattern was found in the classification of laccase gene subfamilies of *C. cinerea* and *P. ostreatus* [51,54]. We observed that laccase genes in the same group had a similar number of introns. For example, *AaLac7* and *AaLac8* are more closely related to the *PoLac2* gene from *P. ostreatus*, and these genes all had complex intron-exon structures [51]. We also found that the structures of *AaLac10* and *AaLac12* were distinct from other laccase genes of *A. areolatum*, and exhibited high homology with the *CcLac16* and *CcLac17* genes from *C. cinerea* [54]. Similarly, these two genes in *C. cinerea* had no intron positions in common with the genes from subfamily 1 and formed subfamily 2 [54]. The exon theory suggested that the number and distribution of introns are related to gene evolution [55]. Thus, the more complex intron-exon structures seen in some of the laccase genes may indicate their later emergence, as over evolutionary time, introns reorder, leading to the fusion of exons to create more complex genes [51,55]. Analysis of the characteristics of the identified *A. areolatum* laccase proteins showed that, except for AaLac3, all laccases have signal peptides and no transmembrane region. The presence of a signal peptide, the absence of a transmembrane domain and a relatively high number of cysteine residues and disulfide bonds indicate that these proteins are likely extracellular in nature [41,56,57].

Three characteristic copper oxidase domains can be used to identify and distinguish laccases within the broader class of multicopper oxidases [50]. Despite differences in the amino acid sequences of the different laccase proteins we identified, there was a high degree of conservation within these domains, suggesting that all of the AaLac proteins identified were typical laccases. Phylogenetic analysis with the laccase genes of *C. cinerea* and *P. ostreatus* also confirmed this result. We also identified the signature sequences (L1–L4), containing 12 amino acid residues acting as the copper ligands, in the laccase proteins from *A. areolatum*. Notably, we found that AaLac10 and AaLac12 contained valine residues within the L2 region, where a conserved cysteine was typically present in classical laccases. Interestingly, the ferroxidases Mco1 and Fet3 also lacked the cysteine residue at this site [51,52]. Analysis of the three-dimensional crystalline structures of the laccase proteins also revealed differences in the AaLac10 and AaLac12 proteins; in all other proteins the substrate binding loops I, II, III, and IV were highly conserved and featured a cysteine residue and either an aspartic or glutamic acid in the small β-hairpin loop B4–B5, which interacted with the organic substrates [54]. However, AaLac10 and AaLac12 were missing these residues. Interestingly, the ferroxidase Mco1 (*Phanerochaete chrysosporium*) also lacks the conserved cysteine and aspartic or glutamic acid residues in this region and has strong ferroxidase, and weak laccase activity [52]. However, the laccases CcLac16 and CcLac17 have similar characteristics with laccase, but no ferroxidase activity [54]. Thus, whether AaLac10 and AaLac12 have both of laccase and ferroxidase activities need further study.

The presence of *A. areolatum* fungus is vital for the survival of *S. noctilio* larvae. Indeed, Madden and Coutts suggested that the first and second instar woodwasp larvae feed extensively on *A. areolatum*, and later on wood colonized by the fungus [16]. Previous studies have shown that laccases may help to degrade lignin by using oxygen as the oxidant [58]. However, understanding of the interaction mechanism between laccase and lignin is still poor. Using molecular docking technology to model the interaction between laccase and lignin model compounds, we observed that the laccases tested bound with the greatest efficiency to lignin tetramers, whilst binding to the lignin monomers was weaker. The average interaction energy of the docked structures during 15–25 ns MD simulation supported this conclusion. This was also consistent with the reported binding efficiencies of laccases from the white-rot fungi *Phlebia brevispora* and *Dichomitus squalens* to lignin model compounds [41]. In addition, we found that the binding orientation of the lignin model compounds inside laccases varied greatly. Monomers and dimer usually bound to the same pocket, while trimer and tetramer bound to the same or different pockets separately. Similarly, Kameshwar et al., found that white-rot fungi *P. brevispora* and *D. squalens* laccases showed the same binding patterns [41]. We speculated that some AaLac proteins have higher binding energies with trimer and tetramer might be caused by steric hindrance. Fortunately, when the substrate is real lignin, the introduction of mediators could solve this steric hindrance problem caused by an oversized substrate [43].

We noted that the lower binding energies were observed between AaLac2, AaLac3, AaLac6, AaLac8, and AaLac10 and the lignin model compounds. At the same time, the RMSD and Rg results of MD simulation indicate that the laccase protein can maintain stability when combined with the lignin model compound. Overexpression of PoLac2 of *P. ostreatus* confirmed that this laccase is involved in the degradation of lignin in cotton-straw and phylogenetic analysis suggested that AaLac1, AaLac7, and AaLac8 are closely related to PoLac2 [51]. Thus, it is likely that AaLac8 plays a role in the degradation of lignin by *A. areolatum*. Further investigation is required to confirm whether the other identified laccases can help degrading lignin in *Pinus* species.

Giardina et al., suggested that the occurrence of two hydrophobic residues PHE460 and ILE452 in the near surroundings of the Cu-T1 contributed to the high redox potential [23]. In addition, some studies showed that the laccases with a PHE residue as an axial ligand of the T1 site copper exhibited high redox potential, while laccases with MET residue showed low redox potential [59,60]. We analyzed the amino acid residues around the Cu-T1 site of AaLac proteins. We found that almost all laccases (except for AaLac1) had hydrophobic residues Ile in the near surroundings of the Cu-T1, and no amino acid residues PHE and MET were observed. Therefore, we speculated that AaLac proteins may have similar redox potential, which may not be the main factor affecting the binding energy between laccase and lignin model compounds. Hydrogen bonding and hydrophobic interaction were found to be implicated in the binding of laccase to substrates. For example, analysis of the binding mode of lignin and three ligninolytic enzymes (laccase, lignin peroxidase, and manganese peroxidase) identified hydrogen bonds, as well as hydrophobic, aromatic-aromatic, hydrophilic-hydrophobic and receptor-acceptor interactions present in each docking complex [61]. Furthermore, molecular docking experiments using laccase from *Trametes versicolor* in complex with phenol and bisphenol A also showed that hydrogen bonds and hydrophobic interactions were formed [62]. The docking results of *T. versicolor* laccase with lignin models (2,6-dimethoxyphenol, ferulic acid, guaiacol, sinapic acid and vanillyl alcohol) showed that hydrophobic interactions instead of hydrogen bonds played an essential role in the docking complexes [43]. Conversely, we found numerous hydrogen bonds present between *A. areolatum* laccases and the lignin model compounds, and these were notably present between the complexes with lower binding energies.

By comparing the regiospecificity of binding to various lignin model compounds on the active site of laccases. We found that the number of amino acid residues involved in hydrogen bonding and hydrophobic interaction in the tetramer-laccase complexes were generally higher than that in the monomer-laccase complexes. This may be one of the reasons for the higher binding efficiency between laccase and the lignin compound tetramer. Several laccase amino acid residues were commonly found to be related to the hydrogen bonding and hydrophobic interaction with lignin model compounds, specifically, ALA, GLN, LEU, PHE, PRO, and SER residues. Besides, we found that these amino acid residues generally had low Cα-RMSF values, indicating that the main amino acid residues in the active site region of the laccase proteins were relatively stable and had low structural flexibility. After reaching equilibrium, the laccase and lignin model compound interaction system was very stable. In agreement with our findings, Awasthi et al. identified 11 amino acids (LEU185, ASP227, ASN229, PHE260, SER285, PHE286, GLY413, ALA414, PRO415, ILE476, and HIS479) involved in the binding of fungal laccase with several lignin model compounds (sinapyl alcohol, dimer, trimer, and tetramer) using molecular docking and dynamics simulation experiments [63]. Furthermore, crystallographic studies with *Melanocarpus albomyces* laccase in complex with lignin model compounds implicated seven amino acids ALA, PRO, GLU, LEU, PHE, TRP, and HIS in laccase-lignin binding [64]. Understanding the interaction between laccase and lignin might provide a reference for designing enzyme mutants that may reduce the efficiency of lignin degradation and, therefore, be of significant importance for protecting *Pinus* species.

In the nutrient-poor xylem of pine trees, *A. areolatum* fungus acts as an external gut for *S. noctilio* larvae by degrading wood xylem tissues [20]. In this study, we identified, cloned, and characterized the *A. areolatum* laccases and investigated their ability to bind to a selection of lignin compounds (monomers, dimers, trimers, and tetramers). We identified several laccases that had low binding energies to all of the lignin model compounds tested. We further explored the molecular nature of the laccase-lignin interactions and found hydrogen bonding and hydrophobic interactions to play important roles in the formation of these complexes. The illustration of interactions between laccases and lignin model compounds are very important. We have provided molecular insights into the interaction between the docked complexes through molecular docking and molecular dynamics simulation methods, which would help further understand the mutualistic relationship between the *S. noctilio* and *A. areolatum*.

## 4. Methods

### 4.1. Determination of Laccase Activity

*A. areolatum* was cultured on Potato Dextrose Agar (PDA) at 4 °C and preserved at the Beijing Key Laboratory for the Forest Pest Control, Beijing Forestry University, Beijing, China. For laccase fermentation, five agar plugs were removed from the outer circumference of a seven-day-old PDA plate and inoculated in 100 mL PDB medium [8]. Laccase activity was quantitated according to a previously published method with slight modifications [65]. *A. areolatum* was grown in PDB medium at 28 °C for 19 days, and its laccase activity was measured every two days. Enzyme activity was assayed by analyzing the variation in absorbance of the 2, 2′-azinobis-3-ethylbenzthiazoline-6-sulphonate (ABTS) at 420 nm. Enzyme solution (40 μL) was incubated with ABTS (1 mmol/liter, 40 μL) in sodium acetate buffer of pH 4.6 for 10 min at 30 °C. One unit of enzyme activity was defined as the amount of laccase required to oxidize 1 μmol ABTS per minute. All assays were carried out in triplicate.

### 4.2. Genome-Wide Identification and Cloning of Laccase Family Genes

To search for laccase proteins in *A. areolatum*, the protein sequences corresponding to laccase in *C. cinerea* and *P. ostreatus* were used as queries using BLASTp [51,54,66]. In addition, we also searched for three HMM profiles (PF00394, PF07731, and PF07732) in the genome of *A. areolatum* using HMMER 3.0 software to identify laccase genes. Subsequently, gene-specific primers of the identified laccase genes were designed with Primer Premier 5.0 software (Table S4) [67]. Target laccase sequences were then amplified using PrimeSTAR HS DNA polymerase (Takara, Dalian, China) under the following conditions: 98 °C for 1 min; then 30 cycles of 98 °C for 10 s, 58 °C for 15 s, and 72 °C for 1 min; and then 72 °C for 10 min for a final extension. The PCR products were recovered using EasyPure Quick Gel Extraction Kit (TransGen, Beijing, China), and the products were ligated into the pEASY-Blunt cloning vector (TransGen, Beijing, China), according to the manufacturers’ instructions. Plasmids were transformed into Trans-T1 competent cells (TransGen, Beijing, China) and sequenced by Ruibiotech (Beijing, China). The sequences of these laccases have been deposited into the GeneBnak database with the accession numbers MT648837-MT648850.

### 4.3. Analysis of the Laccase Sequences

All the candidate laccase sequences were further verified against the Pfam database (https://pfam.xfam.org/) [68] and the Conserved Domain Database (CDD) (https://www.ncbi.nlm.nih.gov/Structure/bwrpsb/bwrpsb.cgi). The laccase protein sequences were aligned with ClustalW, and phylogenetic relationships were analyzed using the neighbor-joining method (pairwise deletion and 1000 bootstrap tests) using MEGA 6.0 [69]. The intron-exon structures of laccase genes were analyzed by aligning their CDS with the genomic sequences. Conserved motifs were identified using MEME (http://meme-suite.org/tools/meme) [70] with the maximum number of motifs being set to four. These analysis results were visualized using TBtools v 0.67361 [71].

The physicochemical characteristics of the AaLac proteins were analyzed using the ExPASy ProtParam tool (https://web.expasy.org/protparam/) [72], which provided the protein molecular weight (kDa), theoretical isoelectric point (pI), the number of negatively(-R)/positively(+R) charged residues, extinction coefficients (EC), instability index (Ii), aliphatic index(Ai) and grand average of hydropathicity (GRAVY) for each protein. The signal peptides were predicted using the online software SignalP 5.0 (http://www.cbs.dtu.dk/services/SignalP/) [73]. The transmembrane regions of AaLac proteins were analyzed by TMHMM 2.0 (http://www.cbs.dtu.dk/services/TMHMM/). DiANNA 1.1 web server (http://clavius.bc.edu/~clotelab/DiANNA/) [74] was used to analyze the number of cysteine residues and predict possible disulfide bonds. The secondary structures were predicted by SOPMA (https://npsa-prabi.ibcp.fr/cgi-bin/npsa_automat.pl?page= psa_sopma.html) [75], and the N-Glycosylation sites of the laccase sequences were predicted using the NetNGlyc 1.0 Server (http://www.cbs.dtu.dk/services/NetNGlyc/) [76]. To analyze the signature sequences (L1–L4) and the substrate binding loops (I–IV) of the identified laccases, the multiple sequence alignment of AaLac proteins was performed using Clustal Omega and then printed and shaded by Boxshade server (https://embnet.vital-it.ch/software/BOX_form.html).

### 4.4. Phylogenetic Analysis

Multi-sequence alignment of all putative AaLac sequences with 17 CciLac and 12 PoLac amino acid sequences was performed using ClustalW in MEGA 6.0 [69]. All conserved sites in these laccases were used to build the phylogenetic tree, which was constructed using the neighbor-joining (NJ) method with 1000 bootstrap replications. The final tree was visualized and edited by in iTOL (https://itol.embl.de/) [77].

### 4.5. Homology Modeling and Validation of Laccase Proteins

We searched potential templates for different laccase proteins in the Protein Data Bank database and the SWISS-MODEL template library. Based on high similarity scores, the optimal crystal structures (Protein Data Bank codes shown in Table 2) were selected as templates [78,79,80,81,82], and homology modelings of AaLac proteins were carried out using the SWISS-MODEL web server (https://swissmodel.expasy.org/) [83]. The GalaxyRene server (http://galaxy.seoklab.org/) [84] was used to optimize the side chain conformation of the modeled AaLac proteins and minimize the energy of each conformation. The final 3D models of laccases were validated using the online server SAVES 5.0 (https://servicesn.mbi.ucla.edu/SAVES/) [85] with the Procheck, ERRAT and Verify3D functions. ProSA (https://prosa.services.came.sbg.ac.at/prosa.php) [86] was used to check for potential errors of these 3D models. In addition, the quality of these models was predicted using ProQ (https://proq.bioinfo.se/ProQ/ProQ.html), which reported two quality measures (LGscore and MaxSub).

### 4.6. Ligand Preparation and Molecular Docking

The lignin model compounds, used for this study were monomers (sinapyl alcohol, coniferyl alcohol and *p*-coumaryl alcohol), dimer (guaiacyl 4-O-5 guaiacyl), trimer (syringyl β-O-4 syringyl β-O-4 sinapyl alcohol), and tetramer (guaiacyl β-O-4 syringyl β-β syringyl β-O-4 guaiacyl). These compounds were selected from the NMR database of lignin and cell wall model compounds [63]. Their structures were sketched with ChemDraw Pro v19.0 and Chem3D Pro v19.0 and saved in protein data bank format. The docking experiment involving laccase proteins and lignin model compounds was carried out using AutoDock v4.2 and AutoDock Vina v1.1.2 [87]. We used AutoDock v4.2 to edit proteins and ligand compounds, adding all hydrogens, merging non-polar hydrogens and computing Gasteiger charges. The outputs were saved in PDBQT format. We used AutoDock Vina to perform the molecular docking of lignin model compounds with laccase proteins with the exhaustiveness setting at 32. The best-fitting ligand conformations were selected based on their minimum binding energies. The interactions (hydrogen bonds and hydrophobic interactions) of AaLac proteins with lignin model compounds were analyzed and depicted with LigPlot+ and PyMol v2.4.0 [88,89].

### 4.7. Molecular Dynamics Simulation of Docked Complexes

Docked complexes of laccases having high binding efficiencies with the lignin model compounds (SA, dimer and tetramer) were selected for MD simulation analysis. Gromacs 2020.3 package [90] was then employed to simulate each complex with Charmm36-mar2019 force field. Transferable interatomic potential with three points model (TIP3P) water molecules were used to solvate the docked complex and Cl^−^ and Na^+^ were added to neutralize the system. Then we performed energy minimization using the steepest descent algorithm to make the maximum energy of the system less than 1000 kJ·mol^−1^·nm^−1^. The minimized system was then heated to 310K and equilibrated for 100 ps in NVT ensemble and another 100 ps in NPT ensemble. Finally, this pre-equilibrated system was subjected to run 25 ns molecular dynamics simulation [63]. All results analysis was performed using Gromacs 2020.3 package.

## Figures and Tables

**Table 1:** The physicochemical properties of laccase proteins in *A. areolatum*.

Gene Name	DNA Length (bp) *	Intron #	Mature Protein (aa)	MW (kDa)	pI	Signal Peptide (aa)	Cysteine Residues #	Predicted S-S Bonds
*AaLac1*	2223	12	523	56.96001	4.70	20–21	5	104–512, 136–474
*AaLac2*	2353	14	517	56.48112	6.12	18–19	5	103–470, 135–224
*AaLac3*	2489	15	518	56.37469	5.04	/	5	106–473, 138–226
*AaLac4*	2182	12	509	54.40684	4.90	20–21	5	104–467, 136–223
*AaLac5*	2910	19	520	55.87515	4.52	22–23	5	107–471, 139–229
*AaLac6*	2579	18	520	56.39443	5.56	20–21	5	/
*AaLac7*	2604	19	524	57.36822	4.81	20–21	5	104–475, 136–230
*AaLac8*	2934	23	529	57.82204	5.04	32–33	5	116–242, 148–480
*AaLac9*	2630	19	525	56.82742	4.93	19–20	5	105–509, 137–226
*AaLac10*	2253	13	520	56.42250	4.53	19–20	3	102–474
*AaLac11*	2717	19	523	57.07710	5.72	22–23	5	107–511, 228–476
*AaLac12*	2332	14	521	56.41483	4.45	19–20	3	102–475
*AaLac13*	2134	11	509	54.50674	4.63	20–21	5	104–467, 136–223
*AaLac14*	2249	13	508	54.90668	4.20	20–21	5	104–467, 136–223

* From ATG to the stop codon; # number.

**Table 2:** Validation of the modeled structures of *A. areolatum* laccases.

Protein Name	Template	Sequence Identity	Coverage	Verify 3D	ERRAT	G-Factors			LGscore	MaxSub	Z-Score	GMQE	QMEAN
						Dihedrals	Covalent	Overall					
AaLac1	3kw7.1.A	53.88%	0.91	90.30%	86.5263	0.12	−0.41	−0.07	4.598	0.313	−7.33	0.75	−3.74
AaLac2	5mhw.1.A	58.37%	0.95	91.89%	83.9248	0.17	−0.37	−0.03	4.995	0.342	−8.45	0.81	−0.24
AaLac3	5ehf.1.A	57.99%	0.94	92.67%	86.8476	0.18	−0.31	0	4.742	0.322	−8.27	0.8	0.08
AaLac4	5mej.1.A	63.39%	0.94	92.07%	80.9829	0.12	−0.38	−0.06	4.544	0.319	−7.94	0.81	−0.98
AaLac5	5mej.1.A	63.69%	0.95	97.17%	82.5	0.16	−0.38	−0.05	5.087	0.343	−7.17	0.81	−0.23
AaLac6	5mhw.1.A	63.37%	0.93	93.25%	89.0985	0.16	−0.42	−0.06	5.022	0.355	−7.16	0.81	0.49
AaLac7	5mew.1.A	52.62%	0.91	92.48%	88.843	0.14	−0.4	−0.06	4.832	0.328	−8.62	0.77	−1.67
AaLac8	5mej.1.A	56.05%	0.89	90.31%	84.8936	0.16	−0.44	−0.06	4.999	0.324	−8.16	0.76	−1.93
AaLac9	5z1x.1.A	60.25%	0.93	95.77%	84.7107	0.14	−0.39	−0.06	4.995	0.345	−7.85	0.79	−1.61
AaLac10	6rhh.1.A	45.67%	0.91	92.02%	85.8672	0.12	−0.42	−0.08	3.891	0.208	−7.4	0.74	−1.52
AaLac11	5ehf.1.A	61.54%	0.92	96.93%	85.2083	0.14	−0.42	−0.06	5.089	0.36	−7.94	0.8	−0.51
AaLac12	2xyb.1.A	42.86%	0.93	92.46%	83.8057	0.13	−0.44	−0.08	4.179	0.233	−7.08	0.72	−3.5
AaLac13	5mew.1.A	65.06%	0.94	93.11%	88.0952	0.14	−0.36	−0.04	4.245	0.29	−7.96	0.81	−0.27
AaLac14	5mew.1.A	62.21%	0.94	95.82%	89.0792	0.14	−0.35	−0.04	4.450	0.31	−8.18	0.82	0.28

## Supplementary Materials

The following are available online at https://www.mdpi.com/1422-0067/21/22/8845/s1.

## Abbreviations

PDB	Potato Dextrose Broth
NJ	Neighbor-joining
PDA	Potato Dextrose Agar PDA
ABTS	2,2′-azinobis-3-ethylbenzthiazoline-6-sulphonate
