# Supplementary material for: Genes Identification, Molecular Docking and Dynamics Simulation Analysis of Laccases from Amylostereum areolatum Provides Molecular Basis of Laccase Bound to Lignin
Source: Int J Mol Sci. 2020 Nov 22;21(22):8845. doi: 10.3390/ijms21228845 (PMC7700495; doi:10.3390/ijms21228845)
Supplement: Supplementary file 1 [file ijms-21-08845-s001.zip › Supplementary Files/Figure S2 Four conserved motifs in A. areolatum laccase proteins analyzed by the MEME search tool..pdf]

Motif1

Motif2

Motif3

Motif4

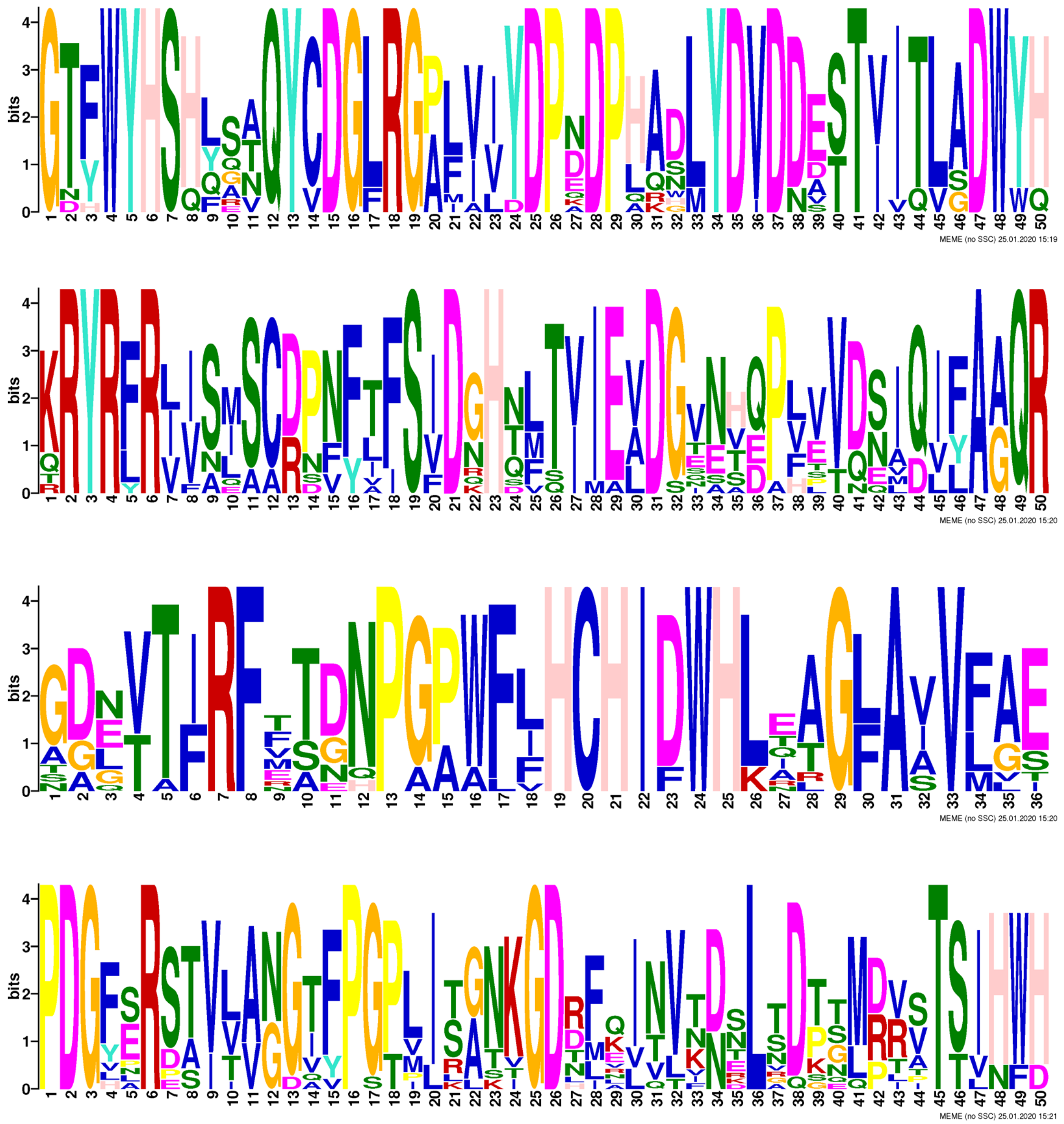

Figure S2 Four conserved motifs in *A. areolatum* laccase proteins analyzed by the MEME search tool. The height of each box represents the specific amino acid conservation in each motif.
