# Supplementary material for: Genes Identification, Molecular Docking and Dynamics Simulation Analysis of Laccases from Amylostereum areolatum Provides Molecular Basis of Laccase Bound to Lignin
Source: Int J Mol Sci. 2020 Nov 22;21(22):8845. doi: 10.3390/ijms21228845 (PMC7700495; doi:10.3390/ijms21228845)
Supplement: Supplementary file 1 [file ijms-21-08845-s001.zip › Supplementary Files/Figure S8 Shows AaLac11 docked with lignin model compounds.pdf]

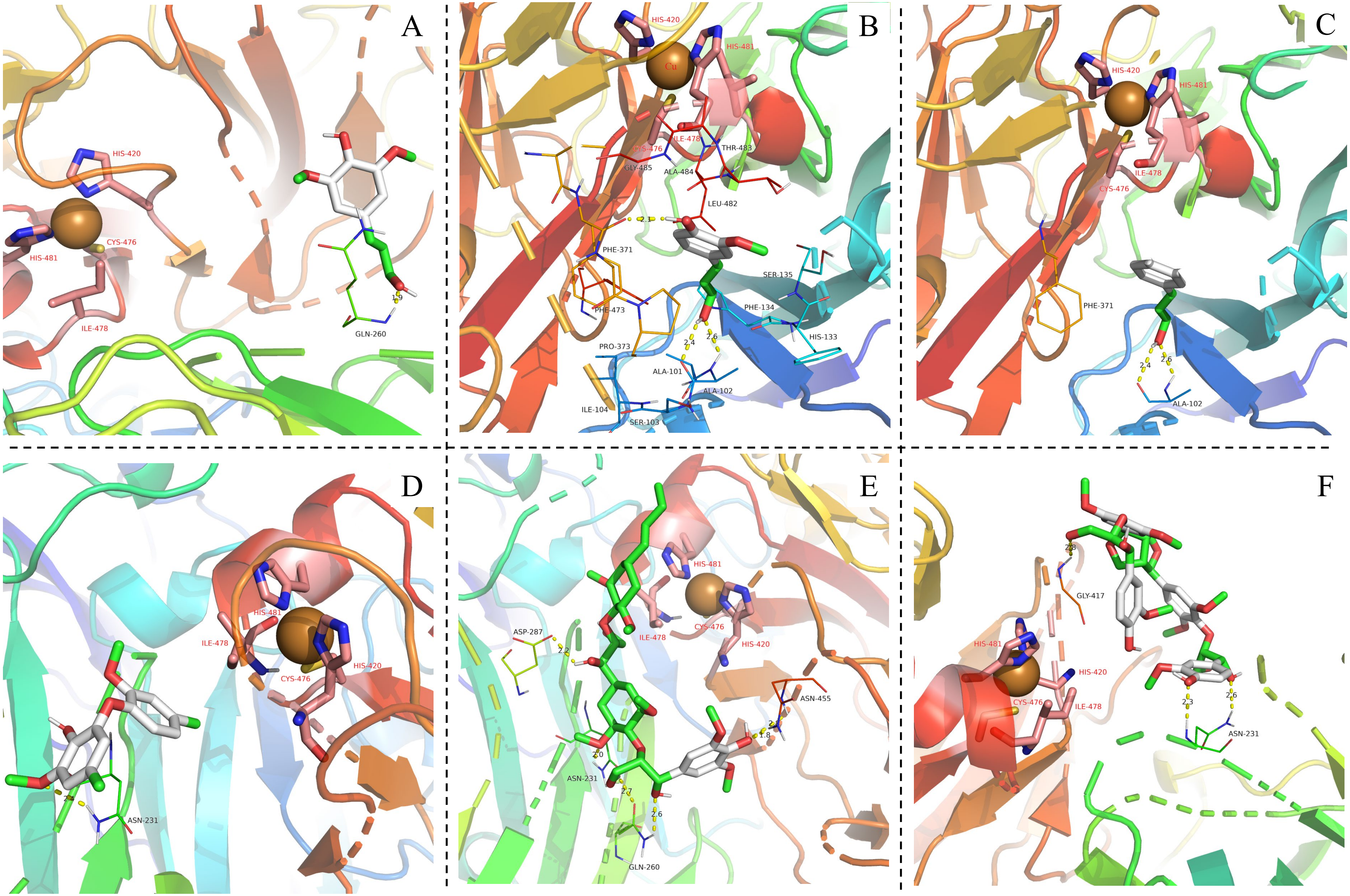


Figure S8 Shows *AaLac11* docked with lignin model compounds. A) Sinapyl alcohol, B) Coniferyl alcohol, C) *p*-Coumaryl alcohol, D) Dimer (guaiacyl 4-O-5 guaiacyl), E) Trimer (syringyl β-O-4 syringyl β-O-4 sinapyl alcohol), F) Tetramer (guaiacyl β-O-4 syringyl β-β syringyl β-O-4 guaiacyl).
