# Supplementary material for: Genes Identification, Molecular Docking and Dynamics Simulation Analysis of Laccases from Amylostereum areolatum Provides Molecular Basis of Laccase Bound to Lignin
Source: Int J Mol Sci. 2020 Nov 22;21(22):8845. doi: 10.3390/ijms21228845 (PMC7700495; doi:10.3390/ijms21228845)
Supplement: Supplementary file 1 [file ijms-21-08845-s001.zip › Supplementary Files/Table S3 The amino acid residues of A. areolatum laccases involved in hydrogen bonding and hydrophobic interaction.docx]

Table S3 The amino acid residues of *A. areolatum* laccases involved in hydrogen bonding and hydrophobic interaction

| Protein | Sinapyl alcohol | Coniferyl alcohol | *p*-Coumaryl alcohol | Guaiacyl 4-O-5 guaiacyl | Syringyl β-O-4 syringyl β-O-4 sinapyl alcohol | Guaiacyl β-O-4 syringyl β-β syringyl β-O-4 guaiacyl |
| --- | --- | --- | --- | --- | --- | --- |
| *AaLac1* | HIS130, SER132, TYR374 | ALA99, HIS130, TYR374, PHE471, LEU480 | ALA99, TYR374, PHE471, LEU480 | PRO98, TYR374, PHE471, LEU480, GLU481 | ARG231, PHE233, TYR264, THR289, ALA419 | VAL187, GLN361, PHE366 |
| *AaLac2* | HIS129, SER131, TYR365 | ALA98, HIS129, SER131, TYR365, LEU467, LEU476, ARG477, GLY479 | ALA98, TYR365, LEU467, LEU476, ARG477 | PHE130, SER131, TYR365 | ASN227, GLN256, ILE284, GLU321, GLY411, ALA429, ASN449 | ASN227, GLY283, PRO318, LEU319, PRO413, ASN449 |
| *AaLac3* | ALA101, PHE368, SER370, LEU479, ALA480, GLY482 | ALA101, GLY134, PHE368, LEU479, ALA480, GLY482 | ALA101, HIS132, PHE368, LEU479 | ARG182, LEU359, ALA480, THR481 | GLY134, ALA180, PHE368, SER370 | GLY134, ALA135, ARG182, PHE368, SER370, TRP477, ALA480 |
| *AaLac4* | SER99, HIS130, PRO364, LEU473, ILE474 | SER99, HIS130, ALA132, PHE362, PRO364, PHE464, LEU473 | SER99, HIS130, PRO364, PHE464, LEU473, ILE474 | HIS130, ALA132, PHE362, PRO364 | VAL181, ASP224, TYR355, GLY408, ALA409, ALA446 | ALA132, SER354, PRO364, ILE474, ALA475 |
| *AaLac5* | PHE103, ARG135, PRO369, TYR510 | SER102, PHE103, HIS133, TYR134, ARG135, PRO369 | SER102, HIS133, ARG135, PHE367, PRO369, PHE468, ASN478 | PRO369, PHE468, LEU477 | PHE103, ARG135, ASN136, PHE367, PRO369, LEU477, ASN478 | ASN232, GLN261, ASP288, VAL413, GLN414, LEU450 |
| *AaLac6* | PHE258, GLN261, TYR263, GLU320, ARG440 | ALA100, HIS131, GLN133, SER366 | TYR172, LEU175, PRO180, TRP472 | ALA100, PHE101, GLN365, SER366, LEU474 | ALA100, GLN133, SER176, GLN365, SER366, PHE465, LEU474, THR475 | ASN227, ASN283, THR284, ALA285, GLU320, ALA409, LEU447, HIS473 |
| *AaLac7* | ALA99, LEU481 | ALA99, SER132, TYR375, PHE472, LEU481 | ALA99, TYR375, PHE472, LEU481 | ALA99, PHE100, SER377, PHE472 | ARG231, PHE233, GLY288, THR289, ALA420 | ARG231, ASP262, TYR264, ASP332, GLY418, ALA420, ASN437, HIS480 |
| *AaLac8* | ARG89, ALA111, HIS142, LEU143, GLU487 | ALA111, PHE477, LEU486 | ALA111, HIS142, TYR380, LEU486 | ARG89, PHE112, LEU143, SER144, TYR380, SER382 | SER144, GLN193, PHE194, PHE195, SER382, GLU487 | SER197, PRO201, ARG243, GLY301, GLN367, GLN425, HIS426, HIS485 |
| *AaLac9* | ASP100, ALA101, TYR132, GLN133, PRO369 | ASP100, HIS131, GLN133, PRO369, PHE471, LEU480, GLU481 | ASP100, HIS131, GLN133, PRO369, PHE471 | ASP100, ALA101, TYR132, PHE367, PRO369, PHE471 | GLN258, PHE260, LEU321, GLU323, GLY416, ALA453, THR454, ASP456 | THR352, THR413, VAL414, ALA415, GLY416, THR454 |
| AaLac10 | ARG74, ASP97, LEU129, PRO373 | ARG74, ASP97, LEU129, SER130, PRO373, GLN481 | ARG74, ASP97, PHE98, LEU129, PRO373 | ARG74, PHE98, GLN128, SER130, TYR371, PRO373, GLN481 | HIS179, GLU181, ALA362, ASP416, TRP478 | GLU181, THR183, GLY184, VAL186, ALA362, ASP417, TRP478, GLN481 |
| *AaLac11* | GLN260, PHE262, GLU325 | ALA102, PHE371, PRO373, LEU482 | ALA102, HIS133, PRO373, PHE473 | ASN231, PHE262, PRO421, ILE478 | ASN231, GLN260, ASP287, ASN455 | ILE184, ASN231, GLN260, ASP287, GLY417, PRO419 |
| *AaLac12* | ARG74, ASP97, LEU130, GLN482 | ASP97, GLN129, TYR372, GLN482 | ASP97, GLN129, TYR372, PHE472, LYS481, GLN482, GLY484 | ARG74, GLY131, TYR372, PHE472, LYS481, ALA486 | VAL187, ASP191, SER293, TRP365 | THR184, VAL187, ARG230, GLN261, SER293, GLU419, ASN436, SER437, ALA456 |
| *AaLac13* | TYR171, ALA178 | ALA132, LEU473, ILE474 | TYR171, ALA178, PRO182 | PHE257, GLU320, SER443 | VAL181, PHE257, ALA409, SER443 | ALA354, PHE362, LEU473 |
| *AaLac14* | GLU132, SER175, ALA178, ALA474 | SER99, HIS130, GLU132, PHE362, PRO364, LEU473 | ALA180, VAL181, PHE350, TRP471 | ALA180, VAL181, THR283, PHE355, TRP471 | ASN226, THR228, GLN255, PHE257, LEU280, GLU320, ALA426 | GLN255, PHE257, GLU320, THR406, GLY408, ALA409, ALA426, ASN428 |

Aminoacids represented in red are involved in hydrogen bonding between protein and ligand; aminoacids represented in black are involved in hydrophobic interaction between protein and ligand; aminoacids represented in blue are involved in both hydrogen bonding and hydrophobic interaction between protein and ligand
