# Supplementary material for: Genes Identification, Molecular Docking and Dynamics Simulation Analysis of Laccases from Amylostereum areolatum Provides Molecular Basis of Laccase Bound to Lignin
Source: Int J Mol Sci. 2020 Nov 22;21(22):8845. doi: 10.3390/ijms21228845 (PMC7700495; doi:10.3390/ijms21228845)
Supplement: Supplementary file 1 [file ijms-21-08845-s001.zip › Supplementary Files/Table S4 A. areolatum laccase gene primers used for PCR analysis.docx]

Table S4 *A. areolatum* laccase gene primers used for PCR analysis

| Gene name | Primer name | Primer sequence (5’-3’) |
| --- | --- | --- |
| *AaLac1* | Fw | ATGGTTTCCCTTACCACCCGT |
|  | Rw | TTACTGGAACTCGGGCTCCAG |
| *AaLac2* | Fw | ATGCGATTGGGATCCCTTCTTCTC |
|  | Rw | TCAAAGAGTTTTCTTCTGAGCGAACCG |
| *AaLac3* | Fw | ATGCGCGCTCCTTCTGC |
|  | Rw | CTACATCTTCTGCGTGTACTTGGTATACG |
| *AaLac4* | Fw | ATGCGTCTCACCCAGTCGT |
|  | Rw | TTATGAACCGATGAAGTTATTGTAAGAGGG |
| *AaLac5* | Fw | ATGGCGTTCAAAACACTCACTAGC |
|  | Rw | TTAGGTATTGCCAGTTGAGTTGATGTACG |
| *AaLac6* | Fw | ATGCTCCCAACGCAGTCATCT |
|  | Rw | TTAGTCAGAACCACCAAGGCCG |
| *AaLac7* | Fw | ATGGGCTTCGTCTCTTTCCCC |
|  | Rw | TCACTGGAACTCCGGCGC |
| *AaLac8* | Fw | ATGGCCGGGGTTGGATTTATCG |
|  | Rw | TCACTGGAATTCGGGAGGGAG |
| *AaLac9* | Fw | ATGTTTGGCCCCTCGCTC |
|  | Rw | TCAACCCTGAGTTACGTTGTTGGC |
| *AaLac10* | Fw | ATGAAGGCTTTCCTCGGATCCG |
|  | Rw | TTACTTCTGGTCATCGGGAAGGGC |
| *AaLac11* | Fw | ATGCTACTTTCCTTACAACGCTCCTC |
|  | Rw | TCATTTACCCTTAGGTCCGTCAAAATTGT |
| *AaLac12* | Fw | ATGAAGTCAACCCTCGGTACTCTC |
|  | Rw | CTACTTCTCGTCCTCAGGAAGCG |
| *AaLac13* | Fw | ATGCGTCTCTCCCAGTCCTTC |
|  | Rw | TTAGGCACCGATGAAGGTGTTGTAT |
| *AaLac14* | Fw | ATGCGCCTTACCCAGACATTCC |
|  | Rw | CTAGGCAATGTAGTCGTTGTAGGTGG |
