# Supplementary figures and images for: Genes Identification, Molecular Docking and Dynamics Simulation Analysis of Laccases from Amylostereum areolatum Provides Molecular Basis of Laccase Bound to Lignin
Source: Int J Mol Sci. 2020 Nov 22;21(22):8845. doi: 10.3390/ijms21228845 (PMC7700495; doi:10.3390/ijms21228845)

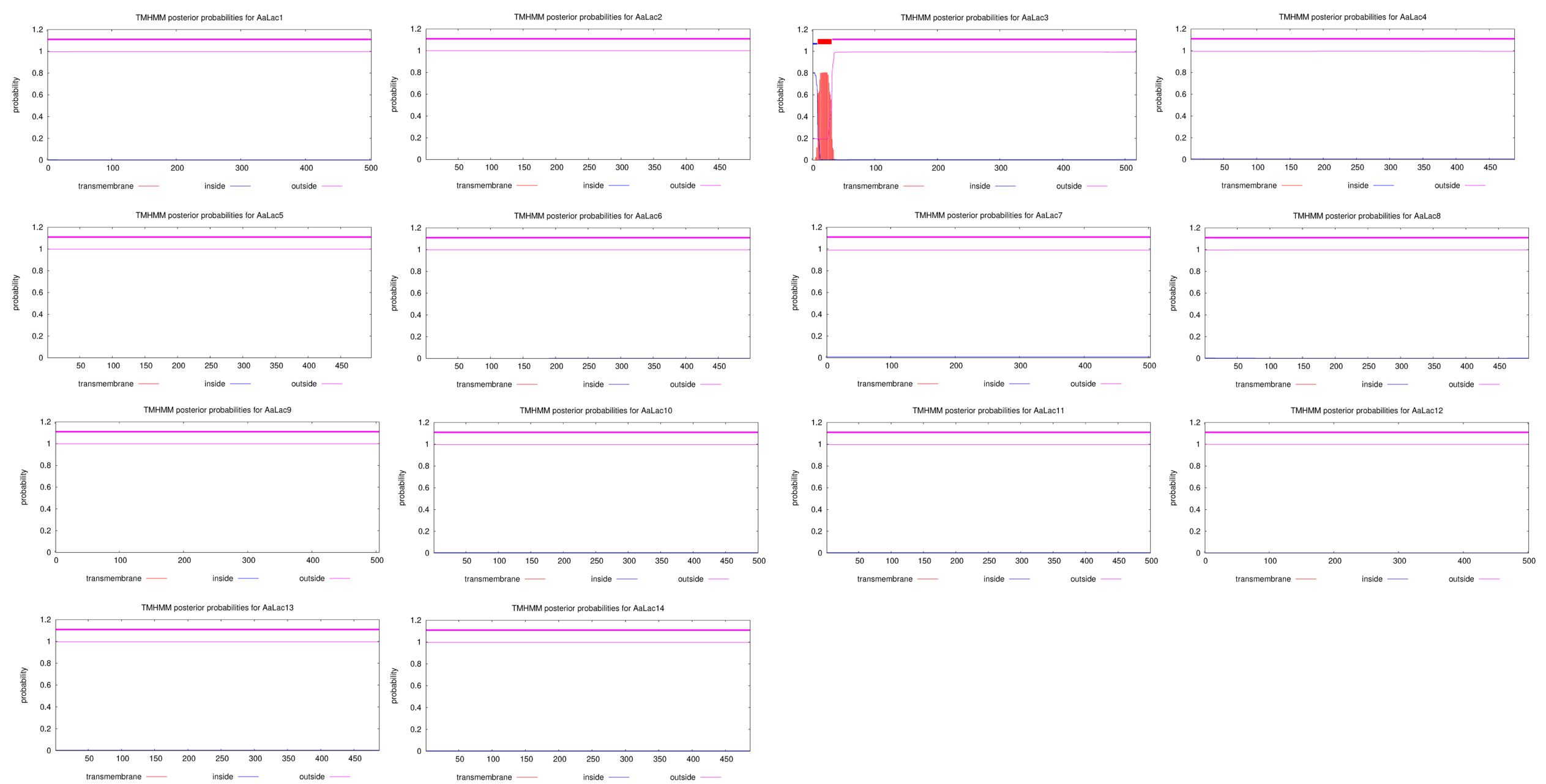

Figure S1 TMHMM plots of *A. areolatum* laccase protein sequences

Supplement: Supplementary file 1 [file ijms-21-08845-s001.zip › Supplementary Files/Figure S1 TMHMM plots of A. areolatum laccase protein sequences.pdf]

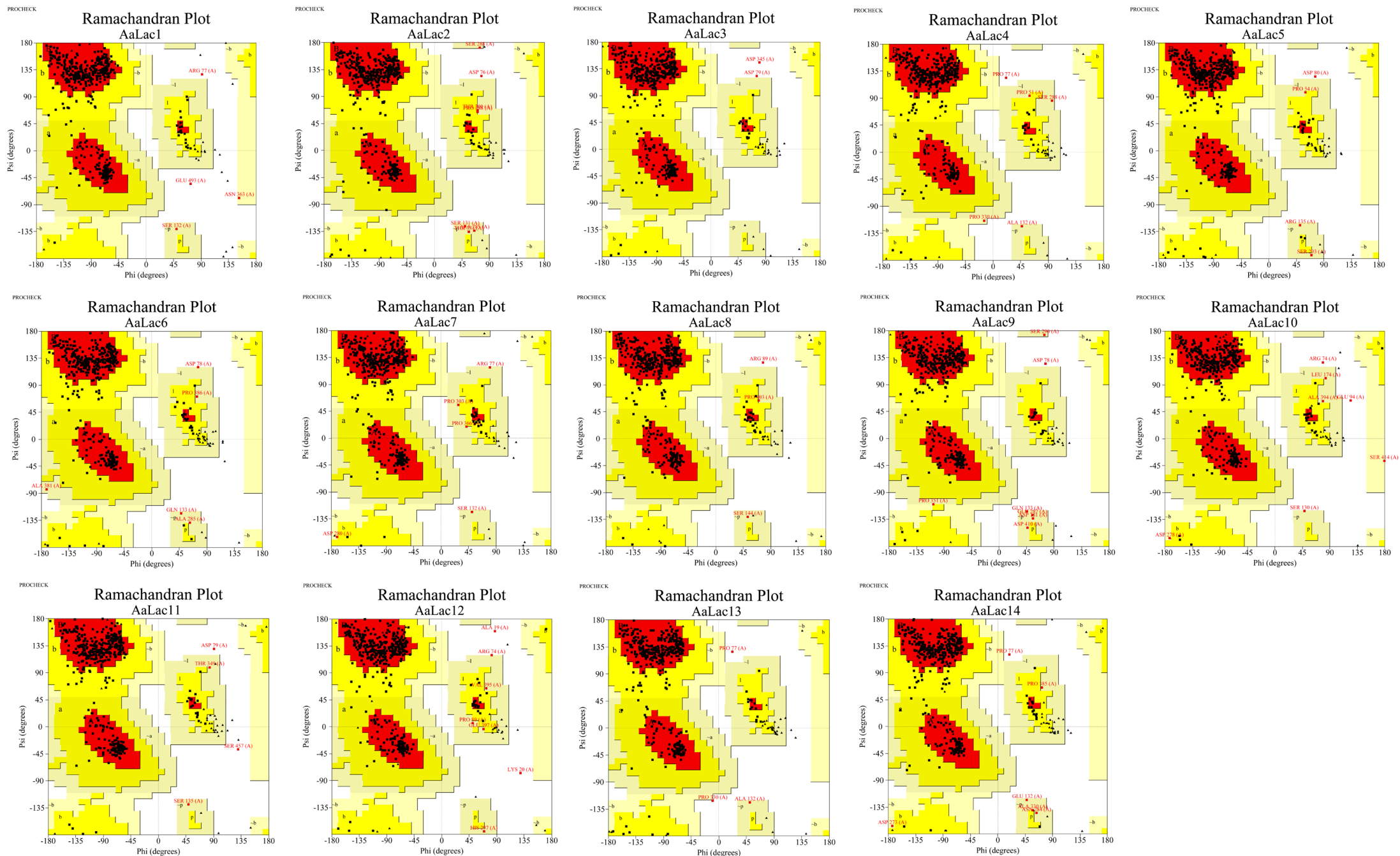

Figure S3 Ramachandran plots of *A. areolatum* laccase protein molecular models

Supplement: Supplementary file 1 [file ijms-21-08845-s001.zip › Supplementary Files/Figure S3 Ramachandran plots of A. areolatum laccase protein molecular models .pdf]
